# Combined Impacts of Physical Activity, Dietary Variety, and Social Interaction on Incident Functional Disability in Older Japanese Adults

**DOI:** 10.2188/jea.JE20210392

**Published:** 2023-07-05

**Authors:** Satoshi Seino, Yu Nofuji, Yuri Yokoyama, Takumi Abe, Mariko Nishi, Mari Yamashita, Miki Narita, Toshiki Hata, Shoji Shinkai, Akihiko Kitamura, Yoshinori Fujiwara

**Affiliations:** 1 Research Team for Social Participation and Community Health, Tokyo Metropolitan Institute of Gerontology, Tokyo, Japan; 2 Integrated Research Initiative for Living Well with Dementia, Tokyo Metropolitan Institute of Gerontology, Tokyo, Japan; 3 Department of Nutrition Sciences, Kagawa Nutrition University, Saitama, Japan; 4 Health Town Development Science Center, Yao City Health Center, Osaka, Japan

**Keywords:** physical activity, dietary variety, social interaction, disability, population-attributable fraction

## Abstract

**Background:**

This 3.6-year prospective study examined combined impacts of physical activity, dietary variety, and social interaction on incident disability and estimated population-attributable fraction for disability reduction in older adults.

**Methods:**

Participants were 7,822 initially non-disabled residents (3,966 men and 3,856 women) aged 65–84 years of Ota City, Tokyo, Japan. Sufficiency of moderate-to-vigorous-intensity physical activity (MVPA) ≥150 min/week, dietary variety score (DVS) ≥3 (median), and social interaction (face-to-face and/or non-face-to-face) ≥1 time/week was assessed using self-administered questionnaires. Disability incidence was prospectively identified using the long-term care insurance system’s nationally unified database.

**Results:**

During a follow-up of 3.6 years, 1,046 (13.4%) individuals had disabilities. Independent multivariate-hazard ratios (HRs) and 95% confidence intervals (CIs) of MVPA, DVS, and social interaction sufficiency for incident disability were 0.68 (95% CI, 0.59–0.78), 0.87 (95% CI, 0.77–0.99), and 0.90 (95% CI, 0.79–1.03), respectively. Incident disability HRs gradually reduced with increased frequency of satisfying these behaviors (any one: HR 0.82; 95% CI, 0.65–1.03; any two: HR 0.65; 95% CI, 0.52–0.82; and all three behaviors: HR 0.54; 95% CI, 0.43–0.69), in an inverse dose-response manner (*P* < 0.001 for trend). Population-attributable fraction for disability reduction in satisfying any one, any two, and all three behaviors were 4.0% (95% CI, −0.2 to 7.9%), 9.6% (95% CI, 4.8–14.1%), and 16.0% (95% CI, 8.7–22.8%), respectively.

**Conclusion:**

Combining physical activity, dietary variety, and social interaction substantially enhances the impacts on preventing disability among older adults, with evidence of an inverse dose-response manner. Improving insufficient behavior elements through individual habits and preexisting social group activities may be effective in preventing disability in the community.

## INTRODUCTION

Maintaining functional ability and preventing disability are important challenges in an aging society. Functional ability is determined by the person’s intrinsic capacity (ie, combination of all physical and mental capacities), relevant environmental factors, and the interaction between the two.^1^ This suggests that only providing preventive services that enhance intrinsic ability, such as physical activity (PA) and/or nutritional programs, is insufficient to promote effective disability prevention. It is also essential to create a social environment in which older adults can improve their physical, mental, and social health through social participation.

There is increasing evidence that combining healthy lifestyle behaviors effectively reduces disability risk.^2^^–^^4^ However, the health behaviors examined previously were limited to behaviors related to the prevention of non-communicable diseases, such as PA (eg, walking), diet (eg, fruit and vegetable consumption), smoking, and alcohol consumption.^2^^,^^3^ In order to prevent frailty and sarcopenia, which are major causes of disability, it is important to also include moderate-to-vigorous-intensity PA (MVPA; eg, muscle strengthening activities) and the consumption of a varied diet containing protein and vitamin D.^5^ There is also growing recognition of the importance of the social aspect of frailty,^6^^,^^7^ with studies reporting that social relationships greatly impact health outcomes, such as disability^8^ and mortality.^9^ Thus, it is necessary to focus on MVPA, dietary variety, and social relationships from the perspective of disability prevention in the community.

To our knowledge, only two observational studies have examined the combined impacts of these behaviors on adverse health outcomes. Although Haider et al^10^ examined the synergistic impacts of the combinations of any two of PA, protein intake, and social network on the development of frailty, they did not investigate the combined impact of all three variables. Pérez-Tasigchana et al^4^ examined the association of six healthy behaviors (not smoking, MVPA, healthy diet, adequate sleeping duration, not being sedentary, and daily social interaction) with incident frailty and disability. However, they failed to include daily social interaction in their analyses of the association between the number of healthy behaviors an individual performs and the incidence of frailty and disability.^4^ Moreover, they did not focus on the combination patterns of the health behaviors. Thus, the combined and public health impacts of MVPA, dietary variety, and social relationships on incident disability are still unclear.

We examined the combined impacts of MVPA, dietary variety, and social interaction on incident disability and estimated the population-attributable fraction (PAF) for disability reduction in community-dwelling older adults.

## METHODS

### Study population

We used data from a community-wide intervention study, launched in 2016, on preventing frailty in Ota City, Tokyo, Japan.^11^^,^^12^ On August 1, 2016, Ota City had a population of 716,645, among whom 162,443 (22.7%) were aged 65 years or older. Of the population aged 65 and older, 15,500 residents (range: 500–4,000 people per district) aged 65–84 years, stratified by sex and age group (65–74 and 75–84 years), were selected using random sampling strategies in all 18 districts of the city.^11^ All participants were physically and cognitively independent, which was defined as the absence of long-term care insurance (LTCI) certification.^13^

**Figure 1.**
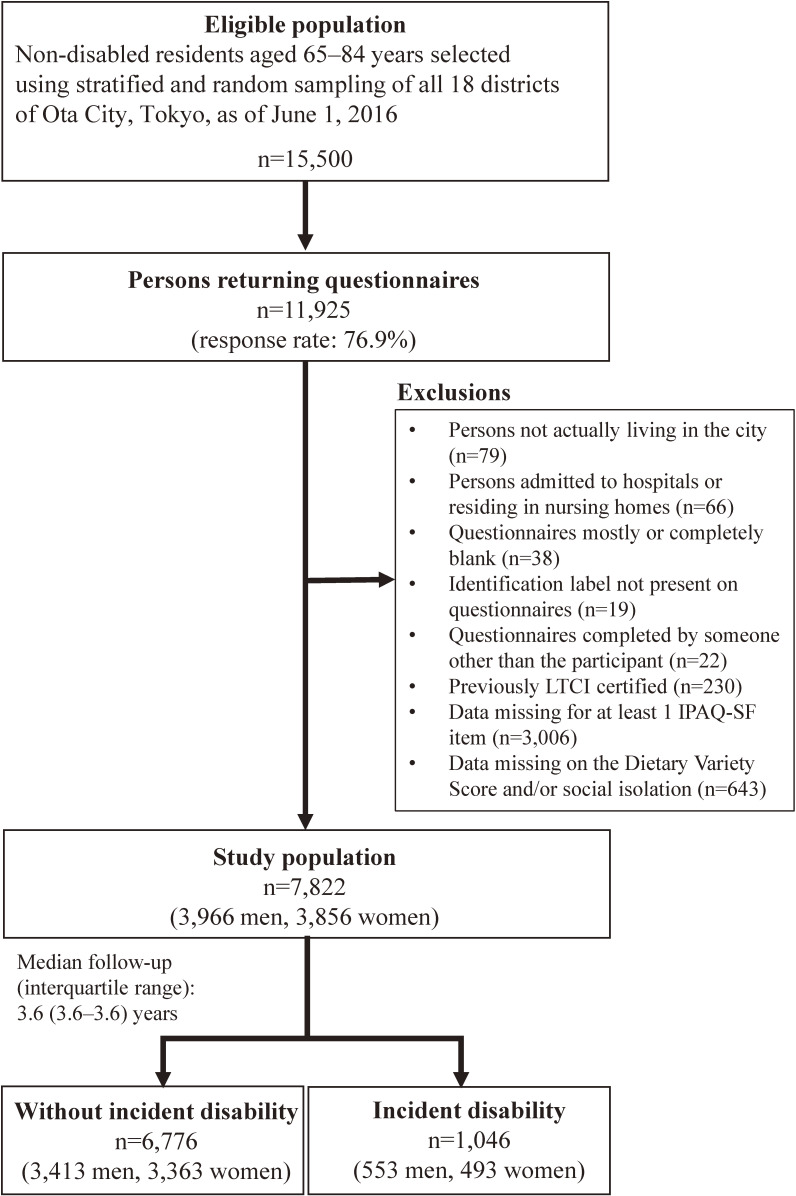
Flow diagram of study participants. IPAQ-SF: International Physical Activity Questionnaire-Short Form; LTCI, long-term care insurance.

Of the 15,500 self-administered questionnaires distributed in July 2016, 11,925 were returned (response rate: 76.9%). The mean district response rate was 77.1% (range: 71.8–80.8%). As Figure 1 shows, 7,822 questionnaires (from 3,966 men and 3,856 women) were included in our analysis. The number of people included in the analysis per district ranged from 214 to 1,920 people (mean: 435).

The study protocol was developed in accordance with the guidelines proposed in the Declaration of Helsinki and was approved by the Ethical Committee of the Tokyo Metropolitan Institute of Gerontology. All participants gave their informed consent before participation.

### Measurements

#### MVPA

We evaluated individuals’ levels of MVPA using the Japanese version of the International Physical Activity Questionnaire-Short Form (IPAQ-SF).^14^^,^^15^ Its external reliability and validity have been previously reported.^14^^,^^15^ Participants were assessed based on their time spent performing vigorous and moderate PA, as well as their walking time during a typical week. Using these values, we defined total MVPA as 7 days × (8.0 metabolic equivalents [METs] × vigorous PA hours/day + 4.0 METs × moderate PA hours/day + 3.3 METs × walking time hours/day).^16^ A total MVPA time ≥150 minutes/week was defined as sufficient PA.^17^

#### Dietary variety

Participants were assessed based on their weekly frequency of consumption of 10 main Japanese food groups (meat, fish/shellfish, eggs, milk, soybean products, green/yellow vegetables, potatoes, fruit, seaweed, and fats/oils). Participants were able to choose from four responses in relation to their consumption of each food group (almost daily, 3 or 4 days a week, 1 or 2 days a week, or hardly ever). A Dietary Variety Score (DVS) was calculated for each participant, using the sum of the number of times each participant answered “almost daily” for each food group (range: 0–10).^18^^,^^19^ Although various DVS cut-off scores (eg, DVS ≥4,^18^^,^^19^ DVS ≥7,^19^ or DVS ≥9^18^) were used in previous studies, these DVS scores came from health check-up data from which DVS scores are typically reported as higher (mean scores ranging from 5 to 7)^18^^,^^19^ than those from self-administered mail survey data (mean scores ranging from 3 to 4).^11^^,^^20^ Therefore, based on the distribution of the study population, the median DVS (score of 3) was used as the cut-off value, and DVS ≥3 was defined as sufficient dietary variety in this study.

#### Social interaction

The level of social interaction was assessed based on the frequency of face-to-face and non-face-to-face (eg, telephone, e-mail, letters) interactions they had with relatives, friends, neighbors, and family who did not live with them. Frequency of contact with others (apart from cohabiting family members) of less than 1 time/week was defined as social isolation.^21^^,^^22^ Thus, sufficient social interaction was defined as contact with others ≥1 time/week.

#### Functional disability

Disability was identified in the participants using the nationally unified database of the LTCI system, enrolment in which is mandatory for all Japanese adults aged 40 years and older. The system provides formal care and support to Japanese adults aged 65 and older with physical and mental disabilities.^13^ LTCI certification is based on a nationally standardized multistep assessment.^13^ Ultimately, the Municipal Certification Committee of Needed Long-Term Care (comprised of physicians, nurses, and health and social service experts) decides whether an older adult should be certified as requiring long-term care and classifies care needs under one of seven levels (support level: 1–2; care level: 1–5).^13^

Disability was defined as the onset of long-term care needs at the support level 1 or above,^23^ using the date of the LTCI application as the date of disability incident. Because new LTCI applications were affected by the novel coronavirus disease pandemic,^24^ we set January 31, 2020 as the endpoint.

#### Covariates

The covariates included age, sex, living situation (living with others or alone), marital status (married, widowed, divorced, or never married), educational attainment (junior high school, high school, or junior college/vocational college/college/graduate school graduate), equivalent income (<2.0, 2.0–3.99, ≥4.0 million yen, or unknown), body mass index (BMI; <18.5, 18.5–24.9, or ≥25 kg/m^2^), alcohol drinking and tobacco smoking statuses (current, never, or former), hypertension, hyperlipidemia, heart disease, stroke, diabetes mellitus, cancer, depressive mood, lower back pain, knee pain, sitting time (<180, 180–299, 300–479, ≥480 minutes/day), and instrumental activities of daily living (IADL) dependence (presence or absence). Equivalent income was calculated by dividing household income by the square root of the number of household members.^25^ BMI was defined as self-rated body weight (kg) divided by self-rated height squared (m^2^). Depressive mood was defined as a score of ≥2 on the 5-item Geriatric Depression Scale.^26^^,^^27^ Sitting time was evaluated using the Japanese version of the IPAQ-SF.^14^^,^^15^ IADL was assessed using the instrumental self-maintenance subscale of the Tokyo Metropolitan Institute of Gerontology Index of Competence,^28^ the total score of which ranges from 0 to 5. Participants whose total score was 5 were classified as independent; those whose total score was ≤4 were classified as dependent.^29^

### Statistical analyses

Data were analyzed using Stata 16.0 (StataCorp, College Station, TX, USA). An α of 0.05 indicated statistical significance. To examine whether minutes/week of MVPA, DVS, and other continuous variables were systematically associated with the number of satisfying health behaviors, we used a trend test (“nptrend” command). Chi-square test compared social interaction and other categorical variables related to the number of satisfying health behaviors.

First, we examined the impact of individual health behaviors on incident disability risk. Because our data had a multilevel structure comprised of individuals (at level 1) nested within 18 districts (at level 2), we used multilevel survival analyses with fixed slopes and random intercept models, calculating adjusted hazard ratios (HR) and 95% confidence intervals (CIs) for incident disability, the dependent variable. Individual health behaviors were included simultaneously as fixed factors. The district was a random factor. We constructed four analytic models. Model 1 was adjusted for age and sex. Model 2 additionally adjusted for living situation, marital status, educational attainment, equivalent income, BMI, hypertension, hyperlipidemia, heart disease, stroke, diabetes mellitus, cancer, alcohol drinking status, and smoking status. Model 3 additionally adjusted for depressive mood, lower back pain, knee pain, and sitting time. Model 4 additionally adjusted for IADL dependency.

Second, the Kaplan-Meier method was used to produce the cumulative probability of remaining disability-free according to the number of satisfying health behaviors. To examine whether the number of satisfying health behaviors was systematically positively associated with the cumulative probability, we used a trend test (“sts test, trend” command).

Third, we performed two analyses: with the number of satisfying health behaviors as the fixed factor and with the combination pattern of health behaviors as the fixed factor. They were included in a dummy form (not satisfying any behaviors was the reference) as fixed factors in the same statistical approach. We tested the statistical interaction between the number of satisfying health behaviors or combination patterns of health behaviors and sex or age group (65–74 or 75–84 years).

Finally, PAFs and their 95% CIs were estimated for individual health behaviors and number of satisfying health behaviors in model 4, using the “punafcc” command.^30^ In the estimation of the PAF of individual health behaviors, we calculated how much the disability rate might have decreased, if participants with insufficient levels of MVPA (<150 minutes/week), DVS (<3), and social interactions (<1 time/week) would have reached the previously defined sufficient level for each behavior. In the estimation of the PAF for the number of satisfying health behaviors, we calculated how much the disability rate might have decreased, if participants with 0 to 2 satisfying health behaviors would have reached sufficient levels for all three health behaviors. The estimations of the PAF when any one or two health behaviors were satisfied were performed in the same manner.

In secondary analyses of the possible influence of reverse causation, we performed sensitivity analyses using the same statistical approach, after excluding individuals who developed disability during the first year of follow-up.

We excluded participants with missing information regarding their disability, IPAQ-SF, DVS, and social interaction from the analysis. The missing covariates were assigned to the “missing” category and included in the analysis to reduce the selection bias.

## RESULTS

During a median follow-up of 3.6 (interquartile range, 3.6–3.6) years, 1,046 participants (13.4%; 553 men and 493 women) presented with disabilities, with rates of 46.3 per 1,000 person-years, and 291 participants (27.8%; 163 men and 128 women) presented with disability during the first year.

Table 1 shows the baseline characteristics of the study population according to the number of satisfying MVPA, dietary variety, and social interaction. Minutes/week of MVPA, DVS, and prevalence of sufficiency of MVPA, DVS, and social interaction were systematically higher with increasing number of satisfying health behaviors (all *P* < 0.001). There were significant differences between groups in sex, marital status, education, equivalent income, BMI, sitting time, and prevalences of hypertension, hyperlipidemia, heart disease, stroke, diabetes mellitus, smoking, depressive mood, lower back pain, and IADL dependency.

**Table 1.** Characteristics of study participants, according to number of satisfying MVPA, dietary variety, and social interaction

Variables		Number of satisfying MVPA, dietary variety, and social interaction^a^				

	All	Not satisfying any behavior	Satisfying any one behavior	Satisfying any two behaviors	Satisfying all three behaviors	*P*-value
	(*n* = 7,822)	(*n* = 451, 5.8%)	(*n* = 1,620, 20.7%)	(*n* = 2,975, 38.0%)	(*n* = 2,776, 35.5%)	
Minutes/week of MVPA, median (interquartile range)	420 (180–840)	0 (0–80)	160 (30–490)	430 (210–900)	570 (350–1,020)	<0.001^b^
MVPA ≥150 minutes/week, *n* (%)	6,156 (78.7)	0 (0.0)	861 (53.2)	2,519 (84.7)	2,776 (100.0)	<0.001
DVS, mean (SD)	3.2 (2.2)	0.9 (0.8)	1.7 (1.6)	2.8 (2.1)	4.8 (1.7)	<0.001^b^
DVS ≥3, *n* (%)	4,457 (57.0)	0 (0.0)	292 (18.0)	1,389 (46.7)	2,776 (100.0)	<0.001
Social interaction of 1 time/week, *n* (%)	5,285 (67.6)	0 (0.0)	467 (28.8)	2,042 (68.6)	2,776 (100.0)	<0.001
Age (years), mean (SD)	73.6 (5.5)	74.0 (5.8)	73.3 (5.6)	73.5 (5.4)	73.8 (5.4)	0.15^b^
Sex (women), *n* (%)	3,856 (49.3)	133 (29.5)	570 (35.2)	1,416 (47.6)	1,737 (62.6)	<0.001
Living alone, *n* (%)	1,535 (19.6)	98 (21.7)	306 (18.9)	569 (19.1)	562 (20.2)	0.22
Marital status, *n* (%)						<0.001
Married	5,327 (68.1)	288 (63.9)	1,104 (68.2)	2,025 (68.1)	1,910 (68.8)	
Widowed or divorced	1,811 (23.2)	103 (22.8)	345 (21.3)	682 (22.9)	681 (24.5)	
Never married	595 (7.6)	53 (11.8)	150 (9.3)	236 (7.3)	156 (5.6)	
Education, *n* (%)						<0.001
Junior high school graduation	1,641 (21.0)	139 (30.8)	460 (28.4)	639 (21.5)	403 (14.5)	
High school graduation	2,946 (37.7)	186 (41.2)	587 (36.2)	1,182 (39.7)	991 (35.7)	
Junior college/vocational college/ ​ college/graduate school graduation	3,034 (38.8)	109 (24.2)	511 (31.5)	1,091 (36.7)	1,323 (47.7)	
Other/missing	201 (2.6)	17 (3.8)	62 (3.8)	63 (2.1)	59 (2.1)	
Equivalent income, *n* (%)						<0.001
<2.0 million yen	1,298 (16.6)	84 (18.6)	291 (18.0)	501 (16.8)	422 (15.2)	
2.0–3.99 million yen	2,739 (35.0)	195 (43.2)	678 (41.9)	1,071 (36.0)	795 (28.6)	
≥4.0 million yen	2,372 (30.3)	127 (28.2)	435 (26.9)	903 (30.4)	907 (32.7)	
Unknown/missing	1,413 (18.1)	45 (10.0)	216 (13.3)	500 (16.8)	652 (23.5)	
Body mass index (kg/m^2^), mean (SD)	22.7 (3.1)	23.2 (3.4)	22.9 (3.3)	22.8 (3.1)	22.4 (2.9)	<0.001^b^
<18.5	609 (7.8)	39 (8.7)	121 (7.5)	208 (7.0)	241 (8.7)	
18.5–24.9	5,563 (71.1)	277 (61.4)	1,113 (68.7)	2,120 (71.3)	2,053 (74.0)	<0.001
≥25	1,606 (20.5)	130 (28.8)	374 (23.1)	631 (21.2)	471 (17.0)	
Hypertension (presence), *n* (%)	4,079 (52.2)	266 (59.0)	898 (55.4)	1,582 (53.2)	1,333 (48.0)	<0.001
Hyperlipidemia (presence), *n* (%)	3,241 (41.4)	197 (43.7)	664 (41.0)	1,227 (41.2)	1,153 (41.5)	0.043
Heart disease (presence), *n* (%)	1,704 (21.8)	123 (27.3)	391 (24.1)	666 (22.4)	524 (18.9)	<0.001
Stroke (presence), *n* (%)	522 (6.7)	50 (11.1)	130 (8.0)	194 (6.5)	148 (5.3)	<0.001
Diabetes mellitus (presence), *n* (%)	1,375 (17.6)	95 (21.1)	314 (19.4)	524 (17.6)	442 (15.9)	0.001
Cancer (presence), *n* (%)	1,266 (16.2)	79 (17.5)	254 (15.7)	510 (17.1)	423 (15.2)	0.08
Alcohol drinking status (current), *n* (%)	4,478 (57.3)	246 (54.6)	949 (58.6)	1,713 (57.6)	1,570 (56.6)	0.73
Smoking status (current), *n* (%)	991 (12.7)	100 (22.2)	310 (19.1)	381 (12.8)	200 (7.2)	<0.001
Depressive mood (presence), *n* (%)	2,564 (32.8)	247 (54.8)	709 (43.8)	978 (32.9)	630 (22.7)	<0.001
Lower back pain (presence), *n* (%)	2,918 (37.3)	190 (42.1)	671 (41.4)	1,131 (38.0)	926 (33.4)	<0.001
Knee pain (presence), *n* (%)	2,352 (30.1)	135 (29.9)	497 (30.7)	933 (31.4)	787 (28.4)	0.20
Sitting time, median (interquartile range)	300 (180–480)	360 (240–600)	300 (180–480)	300 (180–480)	300 (180–480)	<0.001
<180 minutes/day, *n* (%)	1,296 (16.6)	45 (10.0)	258 (15.9)	506 (17.0)	487 (17.5)	<0.001
180–299 minutes/day, *n* (%)	1,751 (22.4)	91 (20.2)	327 (20.2)	682 (22.9)	651 (23.5)	
300–479 minutes/day, *n* (%)	2,031 (26.0)	100 (22.2)	408 (25.2)	800 (26.9)	723 (26.0)	
≥480 minutes/day, *n* (%)	2,276 (29.1)	179 (39.7)	511 (31.5)	811 (27.3)	775 (27.9)	
IADL dependency (presence), *n* (%)	893 (11.4)	130 (28.8)	271 (16.7)	328 (11.0)	164 (5.9)	<0.001

DVS, Dietary Variety Score; IADL, instrumental activities of daily living; MVPA, moderate-to-vigorous physical activity; SD, standard deviation.

^a^Total MVPA time ≥150 minutes/week, DVS ≥3, and social interaction (apart from cohabiting family members) ≥1 time/week were defined as sufficiency, respectively.

^b^*P* for trend test.

The sum of the percentage of respondents and the percentage of missing values (not shown in the table) for each category is 100%.

Table 2 shows independent associations of MVPA, DVS, and social interaction with incident disability. Sufficiency of MVPA (HR 0.68; 95% CI, 0.59–0.78) and DVS (HR 0.87; 95% CI, 0.77–0.99) were independently and significantly associated with lower HRs for incident disability even in the fully adjusted model 4. Although sufficiency of social interaction was significantly associated with lower HR for incident disability up to model 3, the significant association disappeared after adjusting for IADL dependency (model 4: HR 0.90; 95% CI, 0.79–1.03). PAF for disability reduction in satisfying MVPA, DVS, and social interaction were 6.8% (95% CI, 4.8–8.8%), 5.6% (95% CI, 0.7–10.3%), and 3.1% (95% CI, −0.9 to 7.0%), respectively.

**Table 2.** Multilevel survival analyses of independent associations of each MVPA, dietary variety, and social interaction with incident disability (*n* = 7,822)

Variables included simultaneously in the same model	Number of events/ participants	Incidence rate per 1,000 person-years	Model 1			Model 2			Model 3			Model 4			PAF, % (95% CI)	
			
			HR	(95% CI)	*P*	HR	(95% CI)	*P*	HR	(95% CI)	*P*	HR	(95% CI)	*P*	Insufficiency →sufficiency	
MVPA																
Insufficiency	355/1,666	70.1	1.00	(Ref.)		1.00	(Ref.)		1.00	(Ref.)		1.00	(Ref.)		6.8	(4.8–8.8)
Sufficiency	691/6,156	35.8	0.58	(0.51–0.66)	<0.001	0.60	(0.53–0.69)	<0.001	0.63	(0.55–0.72)	<0.001	0.68	(0.59–0.78)	<0.001		
Dietary variety																
Insufficiency	479/3,365	37.8	1.00	(Ref.)		1.00	(Ref.)		1.00	(Ref.)		1.00	(Ref.)		5.6	(0.7–10.3)
Sufficiency	567/4,457	33.4	0.83	(0.73–0.94)	0.004	0.87	(0.76–0.98)	0.030	0.87	(0.77–0.99)	0.037	0.87	(0.77–0.99)	0.034		
Social interaction																
Insufficiency	423/2,537	41.3	1.00	(Ref.)		1.00	(Ref.)		1.00	(Ref.)		1.00	(Ref.)		3.1	(−0.9 to 7.0)
Sufficiency	623/5,285	28.4	0.77	(0.68–0.87)	<0.001	0.81	(0.71–0.93)	0.002	0.86	(0.76–0.99)	0.032	0.90	(0.79–1.03)	0.14		

CI, confidence interval; HR, hazard ratio; IADL, instrumental activities of daily living; MVPA, moderate-to-vigorous intensity physical activity; PAF, population-attributable fraction.

Total MVPA time ≥150 minutes/week, dietary variety (Dietary Variety Score) ≥3, and social interaction (apart from cohabiting family members) ≥1 time/week were defined as sufficiency, respectively.

Model 1: Adjusted for age and sex.

Model 2: Adjusted for variables in model 1, plus living situation, marital status, education, equivalent income, body mass index, hypertension, hyperlipidemia, heart disease, stroke, diabetes mellitus, cancer, alcohol drinking status, and smoking status.

Model 3: Adjusted for variables in model 2, plus depressive mood, lower back pain, knee pain, and sitting time.

Model 4: Adjusted for variables in model 3, plus IADL dependency.

**Figure 2.**
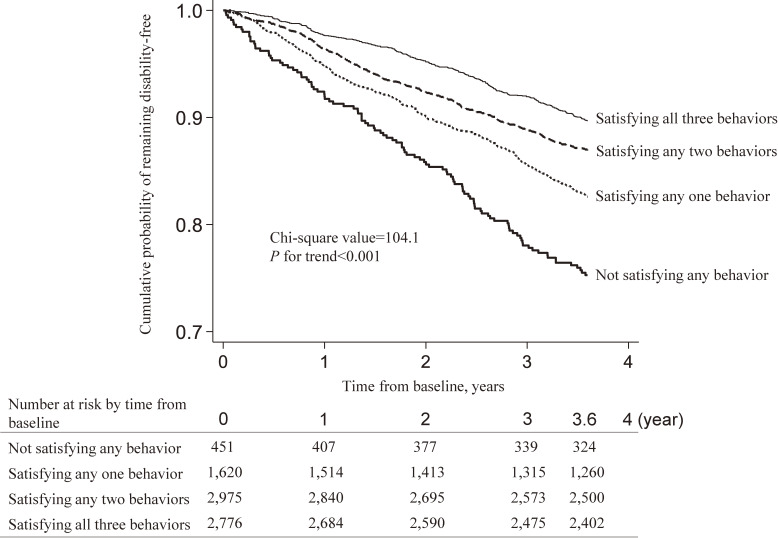
Disability incidence according to number of satisfying MVPA, dietary variety, and social interaction. Total MVPA time ≥150 minutes/week, dietary variety (Dietary Variety Score) ≥3, and social interaction (apart from cohabiting family members) ≥1 time/week were defined as sufficiency, respectively. MVPA, moderate-to-vigorous physical activity.

Figure 2 shows crude cumulative probability of remaining disability-free according to number of satisfying health behaviors. Compared with people who did not satisfy any behaviors, disability risk gradually decreased with increasing number of satisfying behaviors (any one: HR 0.67; 95% CI, 0.53–0.83; any two: HR 0.49; 95% CI, 0.40–0.61; all three: HR 0.38; 95% CI, 0.30–0.47; *P* < 0.001 for trend).

Table 3 shows associations of combination of MVPA, DVS, and social interaction with incident disability. Compared with people who did not satisfy any behaviors, disability risk gradually decreased with increasing number of satisfying behaviors even in model 4 (any one: HR 0.82; 95% CI, 0.65–1.03; any two: HR 0.65; 95% CI, 0.52–0.82; all three: HR 0.54; 95% CI, 0.43–0.69; *P* < 0.001 for trend). PAF for disability reduction in satisfying any one, any two, and all three behaviors were 4.0% (95% CI, −0.2 to 7.9%), 9.6% (95% CI, 4.8–14.1%), and 16.0% (95% CI, 8.7–22.8%), respectively. In the combination pattern, disability risk was consistently and significantly lower in the satisfying only MVPA (HR 0.69; 95% CI, 0.53–0.90), MVPA + dietary variety (HR 0.64; 95% CI, 0.49–0.83), MVPA + social interaction (HR 0.62; 95% CI, 0.48–79), and all three behaviors, (HR 0.54; 95% CI, 0.43–69) even in model 4, as compared with people who did not satisfy any behaviors. The significance of dietary variety + social interaction disappeared in model 4 (HR 0.78; 95% CI, 0.58–1.05; *P* = 0.10). These results did not vary by age group (*P* > 0.24 for all interactions) or sex (*P* > 0.16 for all interactions).

**Table 3.** Multilevel survival analyses of associations of combination of MVPA, dietary variety, and social interactions with incident disability (*n* = 7,822)

Combination categories	Number of events/ participants	Incidence rate per 1,000 person-years	Model 1			Model 2			Model 3			Model 4			PAF, % (95% CI)
			
			HR	(95% CI)	*P*	HR	(95% CI)	*P*	HR	(95% CI)	*P*	HR	(95% CI)	*P*	Insufficiency →sufficiency
Number of satisfying behaviors															
Not satisfying any behaviors	109/451	88.2	1.00	(Ref.)		1.00	(Ref.)		1.00	(Ref.)		1.00	(Ref.)		
Satisfying any one behavior	274/1,620	59.9	0.72	(0.58–0.90)	0.004	0.73	(0.59–0.92)	0.007	0.75	(0.60–0.94)	0.012	0.82	(0.65–1.03)	0.08	4.0 (−0.2 to 7.9)
Satisfying any two behaviors	382/2,975	44.4	0.51	(0.41–0.63)	<0.001	0.55	(0.44–0.68)	<0.001	0.58	(0.47–0.73)	<0.001	0.65	(0.52–0.82)	<0.001	9.6 (4.8–14.1)
Satisfying all three behaviors	281/2,776	34.4	0.38	(0.30–0.47)	<0.001	0.42	(0.34–0.53)	<0.001	0.47	(0.37–0.60)	<0.001	0.54	(0.43–0.69)	<0.001	16.0 (8.7–22.8)
	1,046/7,822	46.3		Trend	<0.001		Trend	<0.001		Trend	<0.001		Trend	<0.001	
Combination pattern of satisfying behaviors															
Not satisfying any behaviors	109/451	88.2	1.00	(Ref.)		1.00	(Ref.)		1.00	(Ref.)		1.00	(Ref.)		
Only MVPA	113/861	45.6	0.60	(0.46–0.78)	<0.001	0.61	(0.46–0.79)	<0.001	0.62	(0.47–0.81)	<0.001	0.69	(0.53–0.90)	0.007	
Only dietary variety	72/292	89.7	0.86	(0.64–1.16)	0.32	0.87	(0.64–1.17)	0.36	0.85	(0.63–1.15)	0.29	0.86	(0.63–1.16)	0.32	
Only social interaction	89/467	68.7	0.83	(0.63–1.10)	0.20	0.87	(0.65–1.15)	0.33	0.91	(0.69–1.22)	0.54	1.02	(0.77–1.36)	0.88	
MVPA + dietary variety	129/933	47.6	0.51	(0.40–0.66)	<0.001	0.55	(0.42–0.71)	<0.001	0.58	(0.44–0.75)	<0.001	0.64	(0.49–0.83)	0.001	
Dietary variety + social interaction	85/456	66.4	0.66	(0.49–0.87)	0.004	0.71	(0.53–0.95)	0.020	0.73	(0.54–0.98)	0.034	0.78	(0.58–1.05)	0.10	
MVPA + social interaction	168/1,586	36.4	0.46	(0.36–0.59)	<0.001	0.49	(0.39–0.63)	<0.001	0.54	(0.42–0.69)	<0.001	0.62	(0.48–0.79)	<0.001	
MVPA + dietary variety + social interaction	281/2,776	34.4	0.38	(0.30–0.47)	<0.001	0.43	(0.34–0.54)	<0.001	0.48	(0.38–0.61)	<0.001	0.54	(0.43–0.69)	<0.001	
	1,046/7,822	46.3													

CI, confidence interval; HR, hazard ratio; IADL, instrumental activities of daily living; MVPA, moderate-to-vigorous intensity physical activity; PAF, population-attributable fraction.

Total MVPA time ≥150 minutes/week, dietary variety (Dietary Variety Score) ≥3, and social interaction (apart from cohabiting family members) ≥1 time/week were defined as sufficiency, respectively.

Model 1: Adjusted for age and sex.

Model 2: Adjusted for variables in model 1, plus living situation, marital status, education, equivalent income, body mass index, hypertension, hyperlipidemia, heart disease, stroke, diabetes mellitus, cancer, alcohol drinking status, and smoking status.

Model 3: Adjusted for variables in model 2, plus depressive mood, lower back pain, knee pain, and sitting time.

Model 4: Adjusted for variables in model 3, plus IADL dependency.

The sensitivity analyses results reveal that the individual association of DVS and incident disability was not robust (see eTable 1). Although the individual association of social interaction and incident disability remained statistically insignificant, it was statistically marginal (HR 0.86; 95% CI, 0.73–1.00; *P* = 0.052). Other results were not substantially different from those of the primary analyses (see eTable 2).

## DISCUSSION

Our study yielded the following findings. First, MVPA was significantly and consistently associated with lower risk of disability in both primary and sensitivity analyses. Second, disability risk gradually decreased with increasing number of satisfying MVPA, DVS, and social interaction in an inverse dose-response manner. Third, if all participants adhered to all three behaviors (except those who satisfied all three behaviors at baseline), 3.6-year disability would be reduced by 16.0%. Finally, these results were not substantially different in the sensitivity analysis, suggesting the importance of combining MVPA, dietary variety, and social interaction in disability prevention efforts in the community.

Our results for each health behavior were consistent with previous studies. Recent studies showed that PA was most protective against frailty^4^ and disability^31^ among traditional and nontraditional health behaviors. There is growing evidence that the Japanese dietary pattern is associated with lower risk of disability,^32^ and dietary variety especially reduces all-cause and cancer mortality.^33^ Although our results on the association of social interaction alone with incident disability were not robust, they were consistent with a recent study.^4^

Interestingly, despite the non-robust associations of DVS and social interaction with incident disability, additional risk reduction was observed when combined with MVPA. Previous studies consistently reported that exercising,^34^^–^^37^ eating,^38^^–^^40^ and doing social activities^35^ with others brought various health benefits compared to doing these activities alone. Evidence of the relationship between this kind of interactive behavior and positive psychological outcomes^39^ is especially robust, and the mechanism can be explained by the psychosocial factors (eg, reducing stress and anxiety and enhancing social connection and support) that mediate the positive effects on mental health.^41^ Moreover, interaction with others not only promotes PA^42^ and dietary variety^43^ but also affects the risk reduction of health outcomes.^9^ By adding a social interaction element, the preventive impact on disability was considered to be reinforced from a psychosocial aspect.

On the contrary, MVPA and dietary variety would enhance the preventive impact on disability from medical and physiological aspects. Previous systematic reviews and meta-analyses of randomized controlled trials showed some positive effects of exercise and nutritional interventions for reducing sarcopenia^44^ and frailty.^45^ In particular, for reducing frailty, a combination of exercise and nutritional supplementation/education—not nutrition only—was found to be effective.^45^ The additive effects on muscle mass, strength, and physical performance may play an important role in preventing frailty and disability. Our results provided further evidence that combining PA and dietary variety with social interaction reduced disability risk and enhanced the public health impact. The clear inverse dose-response association of the number of satisfying MVPA, DVS, and social interaction with disability risk is a noteworthy and important finding of this study.

From our findings and from a public health perspective, we suggest effective countermeasures against disability in the community setting. To combine MVPA, dietary variety, and social interaction, promoting the establishment and continuation of social group activities in the community is a main strategy. Several quasi-experimental studies reported that participation in social activities, such as community salons and self-managed exercise groups, reduced the risk of disability.^46^^,^^47^ Increasing population coverage rates of participation in such groups is an important challenge for achieving population-level disability prevention.

Another important point is adding the insufficient element among MVPA and dietary variety to preexisting social group activities to make it a habit.^12^ This could include adding a 10-minute dietary habit check and/or exchange program to a self-managed exercise group, or a simple muscle strengthening activity and/or the dietary habit check to a cultural group. This may lead to increased diversity of activities and strengthen the preventive functions against disability. Promoting such efforts in collaboration with multiple occupations, institutions, and organizations may be also an effective strategy for preventing disability in the community.

Our study has several limitations. First, the self-administered questionnaire used for measurement might involve recall bias. Although the IPAQ-SF is widely used for the assessment of PA, it tends to overestimate PA, as compared with objective measurement tools.^15^ Moreover, DVS from self-administered questionnaires may be underestimated compared to face-to-face interview surveys.^18^ Although our results were based on the median DVS (of 3) of the study population, desirable DVS is considered to be a higher value.^18^^,^^19^ Thus, our results should be generalized with caution. Second, selection bias is a concern, as people who did not satisfy any behaviors constituted only 5.8% in this study. The exclusion of 3,649 (30.6%) respondents who had missing IPAQ-SF, DVS, or social interaction data may have led to the biased inclusion of a health-conscious population in the analysis. Third, the follow-up period was relatively short. Although our results have been confirmed by a sensitivity analysis, the possibility that undiagnosed medical conditions may have affected health behaviors and/or incident disability cannot be completely excluded. Fourth, an older adult must contact the municipal government to officially certify his or her care needs.^13^ Therefore, some disabled individuals might have failed to report themselves, which may have resulted in the underestimation of disability incidence (detection bias). Finally, it should be noted that this study used data sources from a community-wide intervention study. Although the intervention did not affect population-level frailty at 2 years, the walking time and DVS in the intervention subgroup improved at the population-level.^12^ The present study only assessed baseline health behaviors and was not able to take into account the improvements at 2 years and subsequent changes in these health behaviors during the follow-up. If health behaviors improved further during the subsequent follow-up period after 2 years, the relationships between health behaviors and disability risk shown in this study may have been underestimated.

Despite these limitations, to our knowledge, this study is the first to report the combined impact of MVPA, DVS, and social interaction and its PAF for disability risk. These results are strengthened by the large sample of randomly recruited participants and the high response of 76.9% and follow-up rate of 99.84% (ie, all respondents except people whose survey ID label was removed and some unaddressed people).

### Conclusion

The combination of regular PA, dietary variety, and social interaction substantially enhances the impacts on preventing disability of older adults in an inverse dose-response manner. An approach that adds the insufficient behavior element to individual habits and preexisting social group activities may be effective in preventing disability in a community. A further study will be necessary to confirm the hypothesis in real-world settings.

## SUPPLEMENTARY MATERIAL

eTable 1. Multilevel survival analyses of independent associations of each MVPA, dietary variety, and social interaction with incident disability, excluding individuals who developed disability that occurred during the first year of follow-up (n = 7,531)

eTable 2. Multilevel survival analyses of associations of combinations of MVPA, dietary variety, and social interactions with incident disability, excluding individuals who developed disability that occurred during the first year of follow-up (n = 7,531)

## References

[1] World Health Organization. Global Strategy and Action Plan on Ageing and Health. https://www.who.int/ageing/GSAP-Summary-EN.pdf; 2017 Accessed on August 1, 2021.

[2] Artaud F, Dugravot A, Sabia S, . Unhealthy behaviours and disability in older adults: three-City Dijon cohort study. BMJ. 2013;347:f4240. 10.1136/bmj.f4240 23881930

[3] Zhang S, Tomata Y, Newson RB, . Combined healthy lifestyle behaviours and incident disability in an elderly population: the Ohsaki Cohort 2006 Study. J Epidemiol Community Health. 2018;72:679–684. 10.1136/jech-2018-210464 29627784

[4] Pérez-Tasigchana RF, Sandoval-Insausti H, Donat-Vargas C, . Combined impact of traditional and nontraditional healthy behaviors on frailty and disability: a prospective cohort study of older adults. J Am Med Dir Assoc. 2020;21:710.e1–710.e9. 10.1016/j.jamda.2019.08.025 31636035

[5] Morley JE, Vellas B, van Kan GA, . Frailty consensus: a call to action. J Am Med Dir Assoc. 2013;14:392–397. 10.1016/j.jamda.2013.03.022 23764209 PMC4084863

[6] Lee Y, Chon D, Kim J, . The predictive value of social frailty on adverse outcomes in older adults living in the community. J Am Med Dir Assoc. 2020;21:1464–1469.e2. 10.1016/j.jamda.2020.03.010 32362535

[7] Yamada M, Arai H. Social frailty predicts incident disability and mortality among community-dwelling Japanese older adults. J Am Med Dir Assoc. 2018;19:1099–1103. 10.1016/j.jamda.2018.09.013 30471801

[8] Otsuka T, Tomata Y, Zhang S, . Association between social participation and incident risk of functional disability in elderly Japanese: the Ohsaki Cohort 2006. J Psychosom Res. 2018;111:36–41. 10.1016/j.jpsychores.2018.05.004 29935752

[9] Holt-Lunstad J, Smith TB, Layton JB. Social relationships and mortality risk: a meta-analytic review. PLoS Med. 2010;7:e1000316. 10.1371/journal.pmed.1000316 20668659 PMC2910600

[10] Haider S, Grabovac I, Drgac D, . Impact of physical activity, protein intake and social network and their combination on the development of frailty. Eur J Public Health. 2020;30:340–346. 10.1093/eurpub/ckz191 31665261

[11] Seino S, Kitamura A, Tomine Y, . A Community-wide intervention trial for preventing and reducing frailty among older adults living in metropolitan areas: design and baseline survey for a study integrating participatory action research with a cluster trial. J Epidemiol. 2019;29(2):73–81. 10.2188/jea.JE20170109 29962492 PMC6336723

[12] Seino S, Tomine Y, Nishi M, . Effectiveness of a community-wide intervention for population-level frailty and functional health in older adults: a 2-year cluster nonrandomized controlled trial. Prev Med. 2021;149:106620. 10.1016/j.ypmed.2021.106620 33992656

[13] Tsutsui T, Muramatsu N. Japan’s universal long-term care system reform of 2005: containing costs and realizing a vision. J Am Geriatr Soc. 2007;55:1458–1463. 10.1111/j.1532-5415.2007.01281.x 17767690

[14] Craig CL, Marshall AL, Sjöström M, . International physical activity questionnaire: 12-country reliability and validity. Med Sci Sports Exerc. 2003;35:1381–1395. 10.1249/01.MSS.0000078924.61453.FB 12900694

[15] Lee PH, Macfarlane DJ, Lam TH, . Validity of the International Physical Activity Questionnaire Short Form (IPAQ-SF): a systematic review. Int J Behav Nutr Phys Act. 2011;8:115. 10.1186/1479-5868-8-115 22018588 PMC3214824

[16] The IPAQ group. Guidelines for Data Processing and Analysis of the International Physical Activity Questionnaire (IPAQ)-Short and Long Forms. November 2005. https://docs.google.com/viewer?a=v&pid=sites&srcid=ZGVmYXVsdGRvbWFpbnx0aGVpcGFxfGd4OjE0NDgxMDk3NDU1YWRlZTM; 2005 Accessed on August 1, 2021.

[17] World Health Organization. WHO guidelines on physical activity and sedentary behaviour 2020. https://apps.who.int/iris/rest/bitstreams/1315866/retrieve; 2020 Accessed on August 1, 2021. 33369898

[18] Kumagai S, Watanabe S, Shibata H, . Effects of dietary variety on declines in high-level functional capacity in elderly people living in a community. Nihon Koshu Eisei Zasshi. 2003;50:1117–1124. 14750363

[19] Yokoyama Y, Nishi M, Murayama H, . Dietary variety and decline in lean mass and physical performance in community-dwelling older Japanese: a 4-year follow-up study. J Nutr Health Aging. 2017;21:11–16. 10.1007/s12603-016-0726-x27999844

[20] Yamashita M, Seino S, Nofuji Y, . The Kesennuma study in Miyagi, Japan: study design and baseline profiles of participants. J Epidemiol. 2022;32(12):559–566. 10.2188/jea.JE20200599 33840651 PMC9643787

[21] Fujiwara Y, Nishi M, Fukaya T, . Synergistic or independent impacts of low frequency of going outside the home and social isolation on functional decline: a 4-year prospective study of urban Japanese older adults. Geriatr Gerontol Int. 2017;17:500–508. 10.1111/ggi.12731 26799166

[22] Sakurai R, Yasunaga M, Nishi M, . Co-existence of social isolation and homebound status increase the risk of all-cause mortality. Int Psychogeriatr. 2019;31:703–711. 10.1017/S1041610218001047 30022745

[23] Chen T, Honda T, Chen S, . Dose-response association between accelerometer-assessed physical activity and incidence of functional disability in older Japanese adults: a 6-year prospective study. J Gerontol A Biol Sci Med Sci. 2020;75:1763–1770. 10.1093/gerona/glaa046 32134454 PMC7494030

[24] Seino S, Nofuji Y, Yokoyama Y, . Impact of the first wave of the COVID-19 pandemic on new applications for long-term care insurance in a metropolitan area of Japan. J Epidemiol. 2021;31(6):401–402. 10.2188/jea.JE20210047 33840655 PMC8126680

[25] Organisation for Economic Co-operation and Development. Terms of reference. OECD project on the distribution of household incomes (2017/18 collection). http://www.oecd.org/els/soc/IDD-ToR.pdf; 2017 Accessed August 1, 2021.

[26] Hoyl MT, Alessi CA, Harker JO, . Development and testing of a five-item version of the Geriatric Depression Scale. J Am Geriatr Soc. 1999;47:873–878. 10.1111/j.1532-5415.1999.tb03848.x 10404935

[27] Rinaldi P, Mecocci P, Benedetti C, . Validation of the five-item geriatric depression scale in elderly subjects in three different settings. J Am Geriatr Soc. 2003;51:694–698. 10.1034/j.1600-0579.2003.00216.x 12752847

[28] Koyano W, Shibata H, Nakazato K, . Measurement of competence: reliability and validity of the TMIG Index of Competence. Arch Gerontol Geriatr. 1991;13:103–116. 10.1016/0167-4943(91)90053-S 15374421

[29] Ishizaki T, Kai I, Kobayashi Y, . Functional transitions and active life expectancy for older Japanese living in a community. Arch Gerontol Geriatr. 2002;35:107–120. 10.1016/S0167-4943(02)00002-X 14764349

[30] Newson RB. Attributable and unattributable risks and fractions and other scenario comparisons. Stata J. 2013;13:672–698. 10.1177/1536867X1301300402

[31] Raina P, Ali MU, Joshi D, . The combined effect of behavioural risk factors on disability in aging adults from the Canadian Longitudinal Study on Aging (CLSA). Prev Med. 2021;149:106609. 10.1016/j.ypmed.2021.106609 33984371

[32] Matsuyama S, Zhang S, Tomata Y, . Association between improved adherence to the Japanese diet and incident functional disability in older people: the Ohsaki Cohort 2006 Study. Clin Nutr. 2020;39:2238–2245. 10.1016/j.clnu.2019.10.008 31672331

[33] Otsuka R, Tange C, Nishita Y, . Dietary diversity and all-cause and cause-specific mortality in Japanese community-dwelling older adults. Nutrients. 2020;12:1052. 10.3390/nu12041052 32290256 PMC7230563

[34] Kanamori S, Kai Y, Kondo K, . Participation in sports organizations and the prevention of functional disability in older Japanese: the AGES Cohort Study. PLoS One. 2012;7:e51061. 10.1371/journal.pone.0051061 23226458 PMC3511372

[35] Takeda F, Noguchi H, Monma T, . How possibly do leisure and social activities impact mental health of middle-aged adults in Japan?: an evidence from a national longitudinal survey. PLoS One. 2015;10(10):e0139777. 10.1371/journal.pone.0139777 26431536 PMC4592232

[36] Kanamori S, Takamiya T, Inoue S, . Frequency and pattern of exercise and depression after two years in older Japanese adults: the JAGES longitudinal study. Sci Rep. 2018;8:11224. 10.1038/s41598-018-29053-x 30046117 PMC6060146

[37] Seino S, Kitamura A, Tomine Y, . Exercise arrangement is associated with physical and mental health in older adults. Med Sci Sports Exerc. 2019;51:1146–1153. 10.1249/MSS.0000000000001884 30694973 PMC6553972

[38] Kuroda A, Tanaka T, Hirano H, . Eating alone as social disengagement is strongly associated with depressive symptoms in Japanese community-dwelling older adults. J Am Med Dir Assoc. 2015;16:578–585. 10.1016/j.jamda.2015.01.078 25687929

[39] Tani Y, Sasaki Y, Haseda M, . Eating alone and depression in older men and women by cohabitation status: the JAGES longitudinal survey. Age Ageing. 2015;44:1019–1026. 10.1093/ageing/afv145 26504120 PMC4621239

[40] Tani Y, Kondo N, Takagi D, . Combined effects of eating alone and living alone on unhealthy dietary behaviors, obesity and underweight in older Japanese adults: results of the JAGES. Appetite. 2015;95:1–8. 10.1016/j.appet.2015.06.005 26116391

[41] Kanamori S, Takamiya T, Inoue S. Group exercise for adults and elderly: determinants of participation in group exercise and its associations with health outcome. J Phys Fitness Sports Med. 2015;4:315–320. 10.7600/jpfsm.4.315

[42] Lindsay Smith G, Banting L, Eime R, . The association between social support and physical activity in older adults: a systematic review. Int J Behav Nutr Phys Act. 2017;14:56. 10.1186/s12966-017-0509-8 28449673 PMC5408452

[43] Nakamura H, Nakamura M, Okada E, . Association of food access and neighbor relationships with diet and underweight among community-dwelling older Japanese. J Epidemiol. 2017;27(11):546–551. 10.1016/j.je.2016.12.016 28629703 PMC5608593

[44] Yoshimura Y, Wakabayashi H, Yamada M, . Interventions for treating sarcopenia: a systematic review and meta-analysis of randomized controlled studies. J Am Med Dir Assoc. 2017;18:553.e1–553.e16. 10.1016/j.jamda.2017.03.019 28549707

[45] Macdonald SH, Travers J, Shé ÉN, . Primary care interventions to address physical frailty among community-dwelling adults aged 60 years or older: a meta-analysis. PLoS One. 2020;15:e0228821. 10.1371/journal.pone.0228821 32032375 PMC7006935

[46] Hikichi H, Kondo N, Kondo K, . Effect of a community intervention programme promoting social interactions on functional disability prevention for older adults: propensity score matching and instrumental variable analyses, JAGES Taketoyo study. J Epidemiol Community Health. 2015;69:905–910. 10.1136/jech-2014-205345 25888596 PMC4552922

[47] Yamada M, Arai H. Self-management group exercise extends healthy life expectancy in frail community-dwelling older adults. Int J Environ Res Public Health. 2017;14(5):531. 10.3390/ijerph14050531 28505140 PMC5451982

