# Supplementary material for: Combined Impacts of Physical Activity, Dietary Variety, and Social Interaction on Incident Functional Disability in Older Japanese Adults
Source: J Epidemiol. 2023 Jul 5;33(7):350–9. doi: 10.2188/jea.JE20210392 (PMC10257989; doi:10.2188/jea.JE20210392)
Supplement: Supplementary file 1 [file je-33-350-s001.pdf]

eTable 1. Multilevel survival analyses of independent associations of each MVPA, dietary variety, and social interaction with incident disability, excluding individuals who developed disability that occurred during the first year of follow-up (n=7,531)

Variables included simultaneously in the same model	Number of events/ participants	Incidence rate per 1,000 person-years	Model 1			Model 2			Model 3			Model 4			PAF, % (95% CI)	
			HR	(95% CI)	*P*	HR	(95% CI)	*P*	HR	(95% CI)	*P*	HR	(95% CI)	*P*	Insufficiency →sufficiency	
MVPA																
Insufficiency	244/1,555	49.3	1.00	(Ref.)		1.00	(Ref.)		1.00	(Ref.)		1.00	(Ref.)		6.7	(4.5–9.0)
Sufficiency	511/5,976	26.7	0.60	(0.51–0.70)	<0.001	0.61	(0.52–0.72)	<0.001	0.64	(0.55–0.75)	<0.001	0.67	(0.57–0.79)	<0.001		
Dietary variety																
Insufficiency	329/3,215	26.4	1.00	(Ref.)		1.00	(Ref.)		1.00	(Ref.)		1.00	(Ref.)		2.0	(-4.4 to 8.0)
Sufficiency	426/4,316	25.3	0.90	(0.78–1.05)	0.17	0.95	(0.82–1.11)	0.51	0.95	(0.82–1.11)	0.55	0.95	(0.82–1.11)	0.54		
Social interaction																
Insufficiency	301/2,415	29.9	1.00	(Ref.)		1.00	(Ref.)		1.00	(Ref.)		1.00	(Ref.)			
Sufficiency	454/5,116	21.0	0.76	(0.66–0.89)	< 0.001	0.79	(0.68–0.93)	0.003	0.83	(0.71–0.97)	0.020	0.86	(0.73–1.00)	0.052	4.6	(0.2–8.9)

CI, confidence interval; HR, hazard ratio; IADL, instrumental activities of daily living; MVPA, moderate-to-vigorous intensity physical activity; PAF, population-attributable fraction.

Total MVPA time ≥150 minutes/week, dietary variety (Dietary Variety Score) ≥3, and social interaction (apart from cohabiting family members) ≥1 time/week were defined as sufficiency, respectively.

Model 1: Adjusted for age and sex.

Model 2: Adjusted for variables in model 1, plus living situation, marital status, education, equivalent income, body mass index, hypertension, hyperlipidemia, heart disease, stroke, diabetes mellitus, cancer, alcohol drinking status, and smoking status.

Model 3: Adjusted for variables in model 2, plus depressive mood, lower back pain, knee pain, and sitting time.

Model 4: Adjusted for variables in model 3, plus IADL dependency.

eTable 2. Multilevel survival analyses of associations of combinations of MVPA, dietary variety, and social interactions with incident disability, excluding individuals who developed disability that occurred during the first year of follow-up (n=7,531)

Combination categories	Number of events/ participants	Incidence rate per 1,000 person-years	Model 1			Model 2			Model 3			Model 4			PAF, % (95% CI)	
			HR	95% CI	*P*	HR	95% CI	*P*	HR	95% CI	*P*	HR	95% CI	*P*	Insufficiency →sufficiency	
Number of satisfying behaviors																
Not satisfying any behaviors	73/415	60.8	1.00	(Ref.)		1.00	(Ref.)		1.00	(Ref.)		1.00	(Ref.)			
Satisfying any one behavior	190/1,536	42.3	0.73	(0.56–0.96)	0.025	0.75	(0.57–0.98)	0.036	0.75	(0.57–0.98)	0.038	0.80	(0.61–1.06)	0.12	4.2	(-0.7 to 8.8)
Satisfying any two behaviors	275/2,868	32.3	0.53	(0.41–0.69)	<0.001	0.56	(0.43–0.73)	<0.001	0.59	(0.45–0.77)	<0.001	0.64	(0.49–0.84)	0.001	9.5	(3.9–14.8)
Satisfying all three behaviors	217/2,712	26.8	0.41	(0.32–0.54)	<0.001	0.46	(0.35–0.61)	<0.001	0.50	(0.38–0.66)	<0.001	0.56	(0.42–0.74)	<0.001	14.0	(5.1–22.0)
	755/7,531	33.8		Trend	<0.001		Trend	<0.001		Trend	<0.001		Trend	<0.001		
Combination pattern of satisfying behaviors																
Not satisfying any behaviors	73/415	60.8	1.00	(Ref.)		1.00	(Ref.)		1.00	(Ref.)		1.00	(Ref.)			
Only MVPA	79/827	32.3	0.60	(0.44–0.83)	0.002	0.61	(0.44–0.84)	0.003	0.61	(0.44–0.84)	0.003	0.66	(0.48–0.92)	0.013		
Only dietary variety	52/272	66.6	0.93	(0.65–1.33)	0.70	0.95	(0.67–1.37)	0.80	0.93	(0.65–1.33)	0.68	0.94	(0.66–1.35)	0.73		
Only social interaction	59/437	46.6	0.81	(0.58–1.15)	0.24	0.84	(0.60–1.19)	0.33	0.86	(0.61–1.21)	0.41	0.95	(0.67–1.33)	0.76		

MVPA + dietary variety	97/901	36.2	0.56	(0.41–0.75)	<0.001	0.60	(0.44–0.81)	0.001	0.62	(0.45–0.85)	0.003	0.67	(0.49–0.92)	0.013
Dietary variety + social interaction	60/431	47.5	0.68	(0.48–0.96)	0.027	0.74	(0.52–1.05)	0.09	0.74	(0.52–1.06)	0.10	0.78	(0.55–1.11)	0.16
MVPA + social interaction	118/1,536	25.9	0.46	(0.34–0.62)	<0.001	0.48	(0.36–0.65)	<0.001	0.52	(0.39–0.70)	<0.001	0.57	(0.42–0.77)	<0.001
MVPA + dietary variety + social interaction	217/2,712	26.8	0.42	(0.32–0.55)	<0.001	0.47	(0.35–0.61)	<0.001	0.50	(0.38–0.67)	<0.001	0.56	(0.42–0.74)	<0.001
	755/7,531	33.8												

CI, confidence interval; DVS, dietary variety score; HR, hazard ratio; IADL, instrumental activities of daily living; MVPA, moderate-to-vigorous intensity physical activity; PAF, population-attributable fraction.

Total MVPA time ≥150 minutes/week, dietary variety (Dietary Variety Score) ≥3, and social interaction (apart from cohabiting family members) ≥1 time/week were defined as sufficiency, respectively.

Model 1: Adjusted for age and sex.

Model 2: Adjusted for variables in model 1, plus living situation, marital status, education, equivalent income, body mass index, hypertension, hyperlipidemia, heart disease, stroke, diabetes mellitus, cancer, alcohol drinking status, and smoking status.

Adjusted for variables in model 2, plus depressive mood, lower back pain, knee pain, and sitting time.

Model 4: Adjusted for variables in model 3, plus IADL dependency.
